# Delay-Aware Reverse Approach for Data Aggregation Scheduling in Wireless Sensor Networks

**DOI:** 10.3390/s19204511

**Published:** 2019-10-17

**Authors:** Dung T. Nguyen, Duc-Tai Le, Moonseong Kim, Hyunseung Choo

**Affiliations:** 1 Department of Electrical and Computer Engineering, Sungkyunkwan University, Suwon 16419, Korea; ntdung@skku.edu (D.T.N.); ldtai@skku.edu (D.-T.L.); 2 Department of Liberal Arts, Seoul Theological University, Bucheon 14754, Korea; moonseong@stu.ac.kr; 3 Department of Computer Science and Engineering, Sungkyunkwan University, Suwon 16419, Korea

**Keywords:** wireless sensor networks, aggregation scheduling, minimum latency

## Abstract

Many time-sensitive applications require data to be aggregated from wireless sensor networks with minimum latency. However, the minimum latency aggregation scheduling problem has not been optimally solved due to its NP-hardness. Most existing ideas rely on local information (e.g., node degree, number of children) to organize the schedule order, hence results in solutions that might be far from optimal. In this work, we propose RADAS: a delay-aware Reverse Approach for Data Aggregation Scheduling that determines the transmissions sequence of sensors in a reverse order. Specifically, RADAS iteratively finds the transmissions starting from the last time slot, in which the last sender delivers data to the sink, down to the first time slot, when the data aggregation begins. In each time slot, RADAS intends to maximize the number of concurrent transmissions, while giving higher priority to the sender with potentially higher aggregation delay. Scheduling such high-priority sender first would benefit the maximum selections in subsequent time slots and eventually shorten the schedule length. Simulation results show that our proposed algorithm dominates the existing state-of-the-art schemes, especially in large and dense networks, and offers up to 30% delay reduction.

## 1. Introduction

Wireless Sensor Networks (WSNs) appear in many industrial applications, ranging from cyber physical systems, environmental monitoring, health care, and especially the emerging Internet of Things (IoT) paradigm [1,2]. The applications require sensors to be spread over a wide area, usually in unattended mode, for a long period of time to collect data. They need to be capable of sensing physical phenomenon, converting sensory data into digital form, and sending the data toward a center base station through multi-hop paths. Such data collection typically deals with energy and delay efficiency requirements. Due to the development of energy harvesting technologies [3] and the advancement in low power radio circuitry [4], the former requirement has been alleviated at a certain degree, but the latter one remains challenging.

Real-time communication matter is in critical need as more and more industrial applications need to respond as fast as possible against any change in the environment [5]. The reason is that the delay may lead to system error, or at least performance degradation [6]. Issues here include multi-hop routing, wireless interference and scarce capacity (due to small form factor). To alleviate such issues, a data aggregation technique has been widely adopted [7,8]. The main idea of aggregation is performing a data combination at intermediate nodes to reduce the number of outgoing packets. This brings three-fold advantages. First, reducing the number of packets lessens the degree of channel contention. Second, the delay of the data collection task could be significantly smaller. Third, transmitting fewer number of packets help the sensors to conserve energy more efficiently, and hence prolong the network lifetime. To date, how fast information can be aggregated to the base station is still under active discussion. Such problem is called Minimum Latency Aggregation Scheduling (MLAS).

The MLAS problem aims at finding a transmission schedule for all nodes, in which the time required to collect data from the whole network is minimized [9]. A scheduling solution for the MLAS problem embraces two procedures: routing structure construction and link scheduling [7]. The former procedure sets up the *sender/receiver* pairs between nodes, and the latter one assigns transmission times to each pair. Two parts of a solution can be performed either sequentially, or simultaneously. In the former procedure, the common routing structures are based on Shortest Path Tree (SPT), Connected Dominating Set (CDS), or Minimum Spanning Tree (MST) [10]. In the latter procedure, the links are scheduled time slot by time slot. In each time slot, a scheduling algorithm schedules a number of transmissions. Such transmissions are determined by a prioritizing metric: which links carry data in the time slot and which stand by. Known prioritizing metrics are based on the number of neighbors, number of children or hop distance to the sink [10,11,12,13,14]. However, those metrics focus on alleviating the effect of collisions in one or two hop neighbors. Therefore, the solutions may fall into local optima.

This paper proposes a Reverse Approach for Data Aggregation Scheduling (RADAS) in WSNs. In lieu of mimicking the flow of data, RADAS sequences the transmissions from the sink down to the farther nodes. To shorten the schedule length, the proposed scheme not only maximizes the number of concurrent transmissions per time slot, but also identifies the sender selections that are more advantageous for subsequent time slots. In order to do that, we combine both link-based and node-based metrics to prioritize the transmissions. Our contribution is three-fold.

- We enumerate the role of each node in a network based on a Breadth First Search (BFS) tree by calculating a novel node metric called Minimum Aggregation Time (MAT). MAT of a node represents the delay lower bound to collect data to it through the BFS tree, and therefore draws useful information about the aggregation load at the node. A node with higher MAT probably should take a later transmission time slot than the smaller MAT ones. Such information can be used to guide the scheduling priority.
- We propose a reverse approach for scheduling that both takes into account the degree of link conflict and the MAT of the sender. While the link conflict degree is helpful to select as many concurrent transmissions in a time slot as possible, MAT helps to be aware of the senders that potentially need more time to aggregate data from their descendants. The algorithm iteratively seeks for a maximum number of concurrent transmissions time slot by time slot, and, at the same time, put the node with higher MAT ahead.
- We conduct an extensive simulation to compare the proposed scheme with the state-of-the-art ideas. We also evaluate the impact of the metrics under various circumstances. Results show that our scheme consistently dominates others at least 20% in terms of aggregation delay, and the peak improvement is almost 30%.

This paper is organized as follows. We present the network model and problem statement in Section 2, then Section 3 reviews the previous works related to the MLAS problem. The main scheme is described in detail in Section 4 with illustrative examples. Section 5 presents the experimental methodology and results. Finally, we conclude the idea in Section 6.

## 2. Network Model and Problem Statement

The network of sensor nodes can be modeled as a containment unit disk graph G=(V,E) in which *V* is a set of vertices (i.e., sensor nodes) including the sink *s*, and *E* is a set of links [15]. Each vertex is a center of its corresponding unit circle, and a link exists between two vertices when one is in the circle of another. We use (u,v) to denote a link between two nodes u,v∈V, that is, *u* and *v* are adjacent, or, equivalently, are neighbors. We assume that if *u* and *v* are neighbors, both (u,v)∈E and (v,u)∈E.

A sensor node can only communicate with neighbors in its communication range, which is normalized to 1 in the unit disk model. Therefore, in large sensor networks, data delivery must be done in a multi-hop manner. Intermediate nodes have to relay data for other nodes, as well as include its data. We target an event-driven sensing environment where every sensor has data and transmits only once in the event period. Data passing through an intermediate node can be aggregated so that all the outgoing data have the same size fitted into one packet, i.e., regardless of how much data a node received from its neighbors, it can aggregate them with its own data into a single outgoing transmission [13]. Such aggregation scheme can be realized by functions like MIN, MAX, SUM, AVERAGE performed on numerical data. For instance, there are smart applications that require monitoring the maximum temperature in a building for fire detection [16]. In this sense, each node can receive data several times but only transmits data once. We use Time Division Multiple Access (TDMA) as a Medium Access Control (MAC) protocol, in which time is slotted and synchronized throughout the entire network [17]. The time to send or receive data can fit into one time slot and the time to aggregate data are also included in the time slot duration [14].

**Figure 1:**
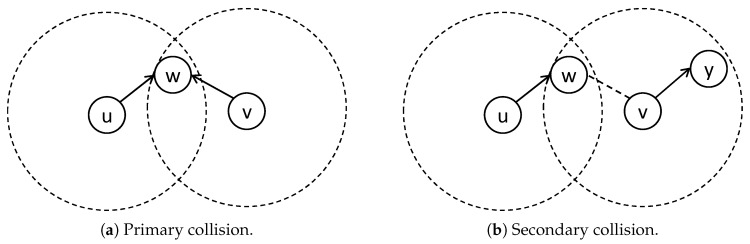
(**a**) Primary and (**b**) Secondary collisions. In both cases, node *w* cannot receive data if nodes *u* and *v* transmit at the same time.

All nodes use the same frequency channel to communicate. The multi-channel model is left for future research. Sensor node cannot transmit and receive data at the same time [7]. When a node is transmitting, all the neighbors can hear the transmission due to the broadcasting nature of wireless transmission. As such, if data transmissions from two or more nodes reach a common node at the same time, a collision occurs at the common node. If the common node is an intended receiver of any transmission, it cannot receive the desired data. There are two types of collisions that should be avoided: primary and secondary [18]. The primary collision happens when two or more nodes simultaneously transmit data to the same intended receiver. As shown in Figure 1a, if nodes *u* and *v* transmit a packet to node *w*, then *w* cannot receive any of the packets. The secondary collision occurs when two nodes intend to transmit data to different receivers at the same time, but one is a neighbor of the other’s receiver. Figure 1b illustrates the case when node *u* transmits data to *w*, and *v* transmits data to *y*; the secondary collision happens at node *w*.

To avoid such collision problem, the sensor nodes need to be scheduled properly. Each node will be assigned a time slot to send data to a specific receiver. Depending on the context, we will say a sender *u* is scheduled to transmit data to a receiver *v* at some time slot *t*, or a link (u,v) is scheduled at time slot *t*. This process is referred to as *link scheduling*, or in short *scheduling*. Once a node is scheduled, it becomes a *child* of its receiver, and its receiver is called its *parent*. The total number of time slots needed to aggregate all data to the sink is *aggregation latency/time/delay* of the network. In this work, we address the MLAS problem, in which the aggregation latency is a minimization objective. The nodes who are supposed to send data to a common receiver must take pairwise different time slots. For the nodes transmitting data in the same time slot, there must be no secondary collision at their intended receivers. Throughout this work, we use notation as in Table 1.

We use *transmission* to refer to a scheduled transmission from a sender to its receiver. We use *physical link* or, in short, *link* to mention the physical communication link that exists between two neighbors. Link (u,v) implies the context that *u* and *v* are neighbors, and are being considered as possible sender and receiver, respectively. When a link (<sender>, <receiver>) is assigned a transmission time slot, we call the sender a *child* and the receiver a *parent*, as eventually such transmissions form an aggregation tree rooted at the sink.

## 3. Related Work

Together with the recent advances in the sensor technologies, the data aggregation problem has been investigated in diverse aspects. For example, the improvement in energy harvesting technology brings new constraint to the MLAS problem [3,19]: the *energy collision*. In another direction, researchers utilize the multi-channel sensors to reduce the channel contention and collision, so as to increase further the number of possible concurrent transmissions and improve the delay efficiency [20,21]. In this problem, the aggregation delay varies depending on the number of available channels. Industrial IoT networks, which necessarily include the WSNs, pose a strict delay requirement to the data aggregation [6] because any delayed aggregation may result in system error. Despite the fact there are variations of MLAS problems, they are still based on the conventional core constraints, which are wireless, have a limited communication range, multi-hop transmissions, and collision avoidance. Such conventional MLAS problem, which is an objective of our work, has attracted a lot of attention [22] and has been approached in different ways, but the improvement lately is insignificant.

A data aggregation protocol consists of two phases: (*i*) routing structure construction; and (*ii*) link scheduling. These phases can be either performed sequentially (phase *i* before phase *ii*) or simultaneously [7]. In the sequential approach, the aggregation scheduling must follow the routing structure determined in the first phase. In the simultaneous approach, the aggregation scheduling process does not depend on any fixed structure; the child–parent pairs are selected during the scheduling. MLAS is known to be NP-hard [23], and, even when the routing structure is given, optimally scheduling the transmissions is an NP-complete problem. In this section, we will brief the status of both directions.

### 3.1. Sequential Approach

In this way, the routing structure construction phase is done separately with the link scheduling phase. Routing structures can be Connected Dominating Set (CDS)-based for earlier works, or tree-based for more recent ones. CDS solutions can guarantee the upper bound of aggregation time, as the time to collect the data for each dominator can be controlled by multiples of Δ, i.e., the maximum degree of a node in the network, and the time to collect data from the dominators to the sink is multiples of *R*, i.e., the network radius [9,24,25]. For example, the authors in [24] proposed an algorithm with a delay upper bound of 4R+2Δ−2, and the scheme in [25] guarantees the delay within 16R+Δ−14. Nevertheless, those upper bounds are too pessimistic [14], and are not helpful in evaluating the effectiveness of a scheme. In addition, because CDS-based structure forces all the dominatees to transmit data to a small number of dominators, a bottleneck of dominators forms, degrading the final aggregation time.

Tree-based solutions usually require categorizing the network nodes into levels such that all nodes in a level have the same hop distance (so-called hop-count) to the sink [13]. Note that one can obtain the hop distance by constructing the Shortest Path Tree (SPT) rooted at the sink and then measure the number of hops from each node to the sink through the tree edges. The criterion to choose a parent for each node is important and seriously affects the final aggregation delay. In some early efforts, nodes in level *L* are only allowed to take a parent in level L−1 [11,13], which will finally result in an SPT. Later, some work allows for forming parent–child relationships between nodes at the same level and even allows for taking a parent in level L+1 [14]. As more flexibility is allowed in the parent selection, it has more potential to get a short aggregation schedule [7]. Specifically, Incel et al. [26] proposed a degree-constrained spanning tree. By considering the number of children as a lower bound for data aggregation scheduling delay, the algorithm prevents the case that nodes near the sink could have a lot of children, causing a bottleneck in the network. They also discussed that, if multiple frequencies are used, then the data collection delay is no longer affected by interference, but the routing tree topology. Malhotra et al. [13] instead considered $\max\{|C\left(u\right)|+d_{u}{\}}_{u\in V}$, in which $d_{u}$ is the depth of node *u* in the aggregation tree as the delay lower bound. They introduced a Balance Shortest Path Tree (BSPT) minimizing their lower bound metric. However, because the routing structure is still purely an SPT, aggregation delay will be undesirably large in high density networks [10]. Pan et al. [14] also constructed a tree named a Minimum Lower bound Spanning Tree (MLST), minimizing the same metric with [13] but relaxing the layering constraint. Each node is free to take its parent in the same, previous, or the next layer with regard to the minimization of the construction metric. A problem of the above-mentioned metric is that it excludes the effect of the whole subtree on the aggregation time of a node. In many cases, the number of children is much less than the actual time required for aggregation.

Regarding the aggregation scheduling phase, a common approach is to assign time slots successively in a bottom–up manner [13,14]. For each time slot *t*, an algorithm first searches for the list of nodes that can be candidate senders. Those nodes are basically the ones that have finished receiving data from its descendants, if any. Then, the candidate senders are examined one by one, and are assigned to transmit at time slot *t* in a first-fit manner. Note that not all of those candidates can be scheduled due to the collisions. The transmissions of selected senders must not cause any collisions with each other. The algorithm terminates when all the links are scheduled and the data finally reach the sink node. For example, WIRES [13] uses the number of non-scheduled neighbors of a node as a ranking priority. In [14], the candidate senders are sorted based on the number of two-hop neighbors. They called their algorithm a Neighbor Degree Ranking (NDR). They also proposed a supplementary scheduling algorithm to shorten the NDR schedule. Such an algorithm allows an unscheduled candidate sender, which cannot be scheduled to transmit to its parent at time *t* by NDR, to flexibly transmit data to another neighbor at time *t*. A common drawback of those scheduling algorithms is that they follow the predefined aggregation trees, which are built ignoring the secondary collisions, so that the solutions cannot guarantee good delay performance.

### 3.2. Simultaneous Approach

**Figure 2:**
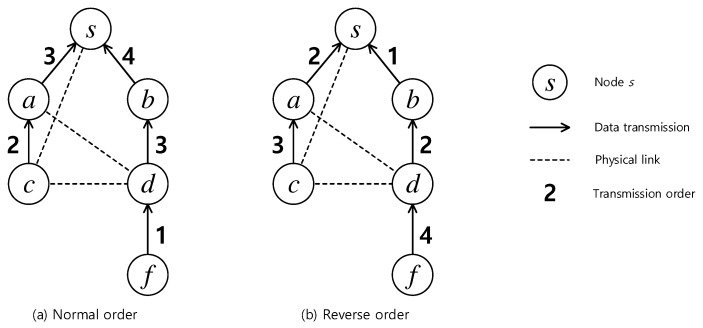
Example of (**a**) a normal order schedule and (**b**) a reverse order schedule.

Simultaneous approach is based on induction theory in which the tree construction is directly guided by the scheduling algorithm. Instead of finding the transmission sequence mimicking the data aggregation flow, a simultaneous approach determines the transmission sequence in a reverse order. The last transmission will be identified first, then the second last transmissions, and so on. Figure 2 shows an example of the normal and reverse order schedules. The last transmission in the normal schedule from node *b* to node *s* is at time slot 4, but, in the reverse schedule, is at time slot 1. Obtaining a normal schedule from a reverse one is therefore straightforward. Within a scope of a time slot, finding a set of concurrent transmissions is a bipartite matching problem [27]. Similar to the sequential approach, the efficiency of a scheme is determined by a prioritization strategy.

There are a very limited number of works following the simultaneous approach. The first work was proposed in [28], which provides an optimal aggregation time for a WSN, but they assume the network is fully connected and ignore the secondary collisions. The authors in [10] claimed that the SPT and CDS-based solutions are neither necessary nor efficient to obtain a small schedule. They identified a set of important nodes in the network: *articulation, pilot* and *critical*, which will definitely take higher priority over other ones during the matching process. After those, the next metric to be considered is node degree and the output algorithm Greedy Growing Tree showed decent improvement over the work done by Malhotra et al. [13]. Lately, Jakob et al. [29], instead of using node-based metrics, use the link–conflict degree to prioritize transmissions. However, their algorithm only resolves the one hop interference, which is not practical in real scenarios. In addition, their algorithm leaves a decent amount of random selection when the link-conflict metric ties among multiple links.

In the sequential approach, the tree construction phase only considers primary collisions, for which it is hard to provide an efficient guideline for the scheduling phase. We avoid this problem by applying the simultaneous approach. In this way of scheduling, each sender–receiver pair and the corresponding transmission time slot are determined on the fly with all collisions considered. Additionally, existing work relies on local information such as number of children, node degree to organize the schedule order, which results in solutions that might be far from optimal. We will show that taking into account the size of a potential subtree rooted at a node will be a better ranking metric in scheduling. The proposed metric *minimum aggregation time* and the scheduling strategy will be presented in the following section.

## 4. Delay-Aware Reverse Approach for Scheduling

As mentioned earlier, a scheduling solution for WSNs consists of two procedures: routing structure construction and link scheduling, which can be performed sequentially or simultaneously. We will discuss the reason that motivates us to go with the simultaneous approach in Section 4.1, and draw the outline of our solution as well. Next, we describe the prioritization metrics in Section 4.2. Finally, in Section 4.3, we detail the use of prioritization metrics to maximize the number of concurrent transmissions in each time slot.

### 4.1. Motivation and Overall Idea

In the sequential approach, the aggregation scheduling must follow the predefined tree and the delay performance varies significantly when the input tree is different [10]. The tree construction is usually performed top–down (by gradually growing the tree starting from the sink node), while the scheduling algorithm assigns a transmission time slot from the leaf nodes up to the sink (i.e., bottom–up). Because it is impossible to foresee the level of secondary collisions during the tree construction, the tree construction and link scheduling phases hardly complement each other. Moreover, existing works try to schedule as many transmissions as possible at the early time slots. Consequently, when going toward the sink nodes, there are more nodes with data ready, but they need to wait for a long time before transmitting. The reason is that the number of receivers reduces very fast around the sink due to collisions.

In contrast, we construct a transmission schedule in a reverse order. Let us assume we already knew the schedule with delay *d*. The basic idea is to find the nodes that transmit data in time slots {d,d−1,…,1}, respectively, and at the same time grow the aggregation tree. It is trivial to see that the transmission in time slot *d* must be directly to the sink from one of the sink’s neighbors. The sink becomes a parent of such sender and, next, in time slot (d−1), they are both the candidate receivers. By assigning the parent–child role, those nodes form a growing tree rooted at the sink and the tree edges are assigned the current time slot. The nodes that are added to the growing tree belong to the *scheduled* set. The remaining nodes are *unscheduled* ones. The process continues until time slot 1. Note that the algorithm iteratively finds as many senders for the candidate receivers in the growing tree as possible, while being fully aware of all collisions between candidate transmissions. The selected senders will join the candidate receiver set in the subsequent iterations. Therefore, the number of candidate receivers are always greedily maximized, which intends to accept the highest number of concurrent transmissions in each time slot. The intuition is that consecutively obtaining the maximal number of concurrent transmissions in every time slot will result in a minimal delay.

**Figure 3:**
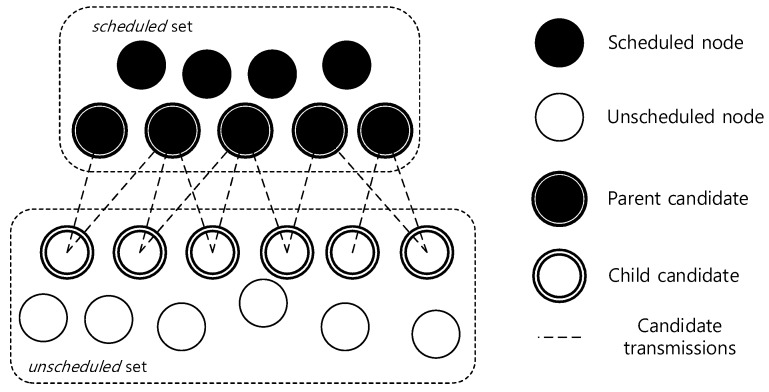
Candidates’ transmissions at a time slot.

Simply put, in each time slot, we are finding a parent–child matching between the nodes in *scheduled* and *unscheduled* sets. Figure 3 shows an example of a bitpartite matching representation in a time slot. The black nodes are scheduled and white nodes unscheduled. The dashed lines are the candidate transmissions between *scheduled* and *unscheduled* sets. Basically, those lines are the physical links between the nodes in two sets. The scheduling algorithm must find a set of links that can transmit data simultaneously without collisions.

Algorithm 1 presents an outline of the reverse order scheduling algorithm. Initially, the scheduled set *S* consists of the sink only, and the unscheduled set *U* contains all the other nodes. Since the final aggregation time *d* is unknown beforehand, the current time slot *t* starts at value 1. Later, at the end of the algorithm, the time slot index will be reversed. Each iteration intends to find the set of links that can transmit data concurrently at time slot *t* (lines 5–14). To do so, it first collects the subset $C_{t}\subseteq U$ consisting of nodes that have links to any node in *S* (line 6). Then, it filters the subset of nodes $P_{t}\subseteq S$ that can become candidate parents of the nodes in $C_{t}$ (line 7). The maximum matching algorithm (line 8) outputs the set of selected links *M*. Based on *M*, it updates the parent–child relationship, assigns the the transmission time slot *t* to each selected child, and updates the set *S* and *U* before proceeding to the next time slot t+1. The obtained transmission order can be easily reverted to a normal schedule, as in lines 16–18.

**Algorithm 1** Reverse Approach for Aggregation Scheduling.Input: G=(V,E)
Output: A time slot assignment for all nodes1:S←{s}2:U←V\{s}3:t←04:**while**S≠V**do**5: t←t+16: $C_{t}\leftarrow \left\{u\in U|N\left(u\right)\cap S\neq \emptyset \right\}$7: $P_{t}\leftarrow \left\{v\in S|N\left(v\right)\cap C_{t}\neq \emptyset \right\}$8: $M\leftarrow \mathrm{find}\;\mathrm{maximum}\;\mathrm{matching}(C_{t},P_{t})$9: **for** (u,v) in *M* **do**10:  p(u)←v11:  timeslot(u)←t12:  S←S∪{u}13:  U←U\{u}14: **end for**15:**end while**16:**for***u* in *V* **do**17: timeslot(u)←t+1−timeslot(u)18:**end for**

To find a maximum matching in the graph $G_{t}=\left(C_{t}\cup P_{t},E_{t}\right)$, in which $E_{t}$ consists of candidate transmissions from the nodes in $C_{t}$ to the nodes in $P_{t}$, the regular maximum bipartite matching solution basically does not work. This is because the matching must deal with the extra collision constraints between links. Unlike in a conventional bipartite matching problem, two links are in conflict if they share an endpoint; under the wireless sensor networks’ environment, conflict can happen between two links with no common endpoint. To deal with such issue, we greedily determine the transmissions one by one, using link-based and node-based metrics as described in subsequent sections.

### 4.2. Scheduling Priority Metrics

#### 4.2.1. Link-Based Metric

**Figure 4:**
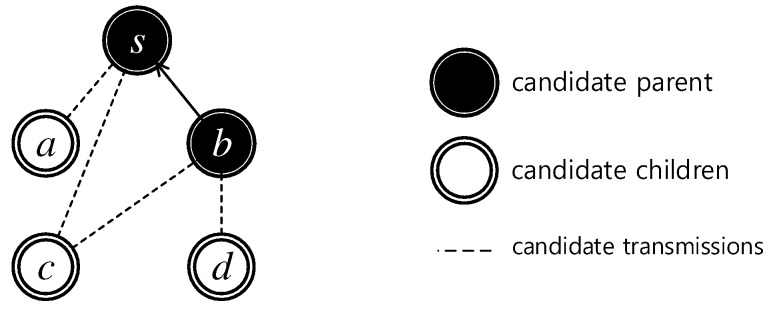
The conflict degrees of candidate transmissions: $l_{L}\left(a,s\right)=2$, $l_{L}\left(c,s\right)=l_{L}\left(c,b\right)=3$ and $l_{L}\left(d,b\right)=2$.

We define a numerical link-based metric named *conflict degree* for the links in the candidate transmissions from $C_{t}$ to $P_{t}$. Two links $\left(u_{0},v_{0}\right)\neq \left(u_{1},v_{1}\right)$ are conflicted if $u_{0}\in N\left(v_{1}\right)$ or $u_{1}\in N\left(v_{0}\right)$. Simply speaking, the conflicted links are either sharing the same sender, or their transmissions cause collisions at one of their receivers. For example, in Figure 4, there are two outgoing links that originated form the sender *c*: (c,s) and (c,b). If we schedule the link (c,s), then *c* intends to deliver the data to *s*. Node *b* does not intend to get the data from *c*; therefore, by assigning the transmission (c,s), we must remove (c,b) from the candidate transmissions. The links (a,s) and (c,s) conflict because *a* and *c* intend to transmit to a common node *s* at the same time, causing primary collision at node *s*. The links (c,s) and (d,b) also conflict since their transmissions cause secondary collision at node *b*. The *link conflict degree* of a link is the number of other links in the candidate transmissions that conflict with the examining link.

Algorithm 2 illustrates the pseudo code to compute the link conflict degree $l_{L}\left(u,v\right)$ with (u,v)∈L. To count the number of links conflicted with (u,v) in *L*, the algorithm needs to iterate through the whole set *L*, which in the worst case can be O(|E|), and, in each iteration, examines the neighbor set of the corresponding nodes. The time complexity is therefore O(|E|Δ). Here, Δ is the maximum degree of the nodes in *G*.

**Algorithm 2** Link Conflict Degree Calculation.Input: set of links L⊆E, link (u,v)∈L
Output: $l_{L}\left(u,v\right)$1:count=02:**for**$\left(u_{0},v_{0}\right)$ in *L* **and** $\left(u_{0},v_{0}\right)\neq \left(u,v\right)$ **do**3: **if** $u_{0}\in N\left(v\right)$ **or** $v_{0}\in N\left(u\right)$ **then**4:  count+=15: **end if**6:**end for**7:**return**count

In Figure 4, nodes {s,b} are scheduled. The rest of the nodes are not scheduled yet, i.e., nodes {a,c,d}. Initially, the link (b,s) is set to transmit data at time slot 1 and the algorithm is finding the links to assign time slot 2. The set of links C={(a,s),(c,s),(c,b),(d,b)}. It is easy to see that $l_{L}\left(a,s\right)=2$ as (a,s) conflicts with {(c,s),(c,b)}. Similarly, $l_{L}\left(c,s\right)=l_{L}\left(c,b\right)=3$ and $l_{L}\left(d,b\right)=2$.

The intuition to minimize the data aggregation delay, as has long been applied, is that, in every time slot, the algorithm tries to maximize the number of concurrent transmissions. This is true for both the sequential and simultaneous approaches. For achieving that goal, one must give higher priority to the link that causes less conflicts to others. However, using link conflict degree would create cases of ties between several links. This number may vary under different network settings, but leaving randomness in the selection will definitely result in a non-optimal solution. For example, in Figure 4, there are two ties between two pairs of links: $l_{L}\left(a,s\right)=l_{L}\left(d,b\right)$ and $l_{L}\left(c,s\right)=l_{L}\left(c,b\right)$. To break the tie among links with a same conflict degree, in this work, we propose a node-based metric, which is calculated per node, as presented in Section 4.2.2.

#### 4.2.2. Node-Based Metric

In the data aggregation scheduling, the importance of a node would be how many descendants the node has to collect data from. Before the transmission of the node is scheduled, it must finish data collection from all the descendants in its subtree, and this information is important to give priority to during the scheduling process. However, in the reverse approach, each node is unable to know how many descendants it may have at the end because the scheduling algorithm grows the aggregation tree from the root to the leaves. Our idea is to first build some simple initial tree rooted at the sink; then, we compute the node metric for each node based on the initial tree.

Evaluating the actual time to collect data through a subtree, with respect to the collision model mentioned in Section 2, is costly. This is because determining a delay optimal schedule even on a given tree is NP-hard [30]. Another way is to use approximation solutions, i.e., heuristic scheduling algorithms [14], but such algorithms are also time-consuming. Our approach, instead, measures the *Minimum Aggregation Time* (MAT), which is the time to collect data to a node from its subtree considering the primary collisions. Computation of MAT is relatively fast, and here we present a MAT calculation when a tree is constructed a priori.

Given a tree, a method to compute the MAT of each node is as follows. Since leaf nodes have no children, their MATs are 0. For non-leaf nodes, their MATs depend on the MATs of their children. Therefore, in order to calculate MAT for a non-leaf node *u*, all the children of *u* must calculate their MATs beforehand. Let $C\left(u\right)=\left\{v_{1},v_{2},\ldots v_{k}\right\}$ be the ordered set of children of node *u* such that:

(1) $$mat\left(v_{i}\right)\leq mat\left(v_{j}\right),\mathrm{where}\;1\leq i<j\leq k.$$

Let $t(v_{i})$ be the transmitting time slot of node $v_{i}$ in a feasible schedule considering primary collision. Because each node only transmits data after it collects all data from its children, we have the following constraint between transmitting time slot and MAT of $v_{i}$:

(2) $$t\left(v_{i}\right)>mat\left(v_{i}\right),\mathrm{where}\;1\leq i<j\leq k.$$

Moreover, the transmitting time slots of the children must be pairwise different:

(3) $$t\left(v_{i}\right)\neq t\left(v_{j}\right),\mathrm{where}\;1\leq i\neq j\leq k.$$

With such constraints, we can formulate the relationship between the MAT and transmitting time of a parent and its children.

**Theorem** **1.** *The minimum aggregation time at a node u is computed as follows:*

(4) $$mat\left(u\right)=\max\{mat\left(v_{i}\right)+k-i+1|1\leq i\leq k\}.$$

**Proof.**  See Appendix A. □

Algorithm 3 depicts the process to calculate MAT for each node in a tree *T*. Let $V^{\prime }$ be the set of nodes whose MATs have not been calculated. Initially, $V^{\prime }=V$ and the algorithm sets 0 to the MATs of all the nodes in $V^{\prime }$ (line 2). Then, it loops through the set $V^{\prime }$ until $V^{\prime }$ becomes empty (line 3). In each loop, it filters out the set *W* consisting of the nodes for which all of their children already had MAT (line 4). The MATs for those nodes in *W* can be computed as in line 6. Finally, it removes the nodes in set *W* from $V^{\prime }$. As the algorithm runs in a bottom–up direction, the last node calculating its MAT is the sink.

**Figure 5:**
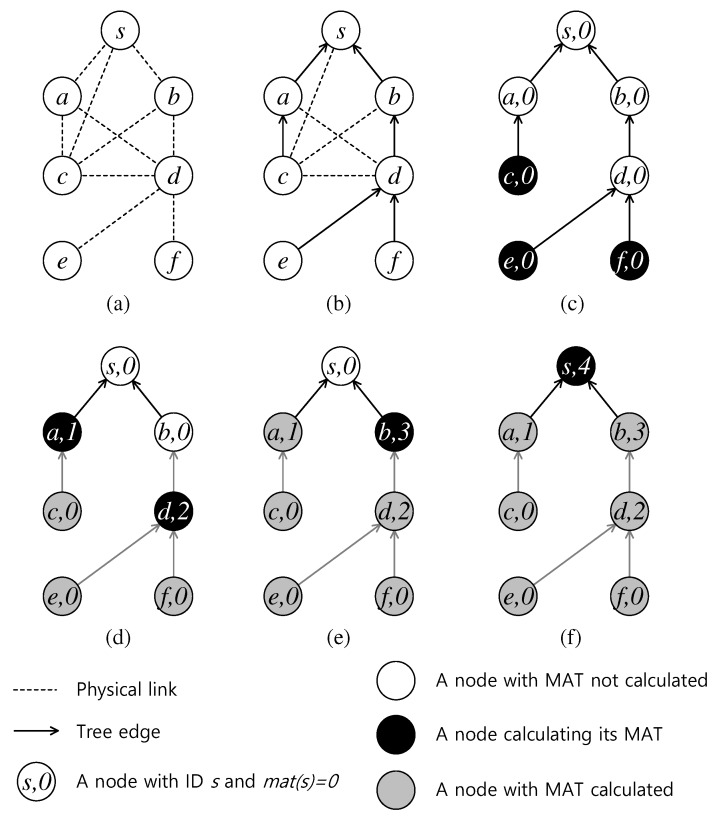
Step-by-step calculation of Minimum Aggregation Time on each node.

Figure 5 illustrates a breakdown in the steps of Algorithm 3 given the network topology Figure 5a and the tree in Figure 5b. First, MAT of all the nodes are set to 0. In Figure 5c, the algorithm examines the leaf nodes {c,e,f}, and the MATs of those nodes remain 0. In the next iteration, nodes *a* and *d* have all of their children with computed MATs (the nodes marked in grey), so they can compute their MATs accordingly. Although two children of node *d*, i.e., nodes *e* and *f*, have equal MAT: mat(e)=mat(f)=0 and they can immediately transmit their data at time slot 1, transmissions of *e* and *f* must take two different time slots. Consequently, 2 is the minimum time that their packets can successfully reach their parent *d*. Similarly, in the next step, only node *b* gets mat(b)=3, and, finally, mat(s)=4 as in Figure 5f.

Calculating MAT for each node requires O(Δ) time due to Theorem 1. Thus, the time complexity of MAT calculation for the whole set of nodes *V* is O(|V|Δ) plus the time to build an SPT using the BFS algorithm, which can be done in O(|V|+|E|). In the whole process, MAT is only computed one time.

**Algorithm 3** Computing Minimum Aggregation Time.Input: A tree *T* built on G=(V,E)
Output: MATs of all nodes in *V*1:$V^{\prime }\leftarrow V$2:mat(v)=0forallv∈V3:**while**$V^{\prime }\neq \emptyset$**do**4: $W\leftarrow \{u\in V^{\prime }|C\left(u\right)\backslash V^{\prime }=\emptyset \}$5: **for** u∈W **do**6:  Compute mat(u) based on Equation (4)7:  $V^{\prime }\leftarrow V^{\prime }\backslash \left\{u\right\}$8: **end for**9:**end while**

### 4.3. Delay-Aware Maximum Matching

Algorithm 4 describes the matching mechanism based on link conflict degree and MAT. Here, we assume that the MAT of all nodes are achieved beforehand by constructing a BFS tree and then applying Algorithm 3 onto the tree. After that, we keep the MAT information of each node, and remove all the parent–child assignment related to the initial BFS tree.

**Algorithm 4** Delay-aware Maximum Matching.Input: G=(V,E); *C*: set of child candidates, *P*: set of parent candidates Output: Set of links without conflict1:L←{(u,v)|u∈C,v∈P,(u,v)∈E}2:M←∅3:**while**L≠∅**do**4: Compute conflict degree for the links in *L*5: $k\leftarrow \min\{l_{L}\left(u,v\right)|\left(u,v\right)\in L\}$6: $K\leftarrow \{\left(u,v\right)\in L|l_{L}\left(u,v\right)=k\}$7: $\left(u_{0},v_{0}\right)\leftarrow \underset{MAT(u)}{arg max}\left\{\left(u,v\right)\in K\right\}$8: $M\leftarrow M\cup \{\left(u_{0},v_{0}\right)\}$9: $L\leftarrow L\backslash \{\left(u_{0},v_{0}\right)\}$10: Remove links that conflicts with $\left(u_{0},v_{0}\right)$ from *L*11:**end while**12:**return***M*

The set of links between *C* and *P* are stored in *L*. The set *M*, which later will contain conflict-free links extracted from *L*, is initialized as ∅ (line 2). Looping through the set *L*, in each iteration, the conflict degree of the links in *L* is refreshed (line 4). Then, the algorithm finds the smallest value of conflict degree *k*, and the set of links that have conflict degree equal to *k* (lines 5–6). Among those links, the one with highest MAT value of the sender will be selected (line 7). By doing so, the algorithm gives priority to the sender that potentially needs more time to collect data from its subtree. Such method would provide a benefit to subsequent iterations. After a link is selected, the algorithm updates *M* and removes the link, and all the conflicted ones out of *L*. Finally, the list of collision-free links *M* will be returned.

Filtering out *L* as in line 1 would take at most O(|V|Δ) time. The algorithm can loop through the list *P* and then check the neighbors of each node in *P* if the neighbor is added to *L* or not. Next, the algorithm turns into a while loop (line 3) and only stops when L=∅. Next, computing conflict degree for each link in *L* takes $O(|L{|}^{2}\Delta )$ (line 4). In lines 5 and 6, filtering out the lowest link conflict degree and collecting all the links with the same link conflict degree in *L* will take O(|L|) time. The next steps, lines 7–10, require no more time than the step in line 4. Thus, Algorithm 4 can be done in $O(|L{|}^{3}\Delta )$. The cardinality of *L* is bounded by |E|; therefore, the time complexity of Algorithm 4 is $O(|E{|}^{3}\Delta )$.

**Figure 6:**
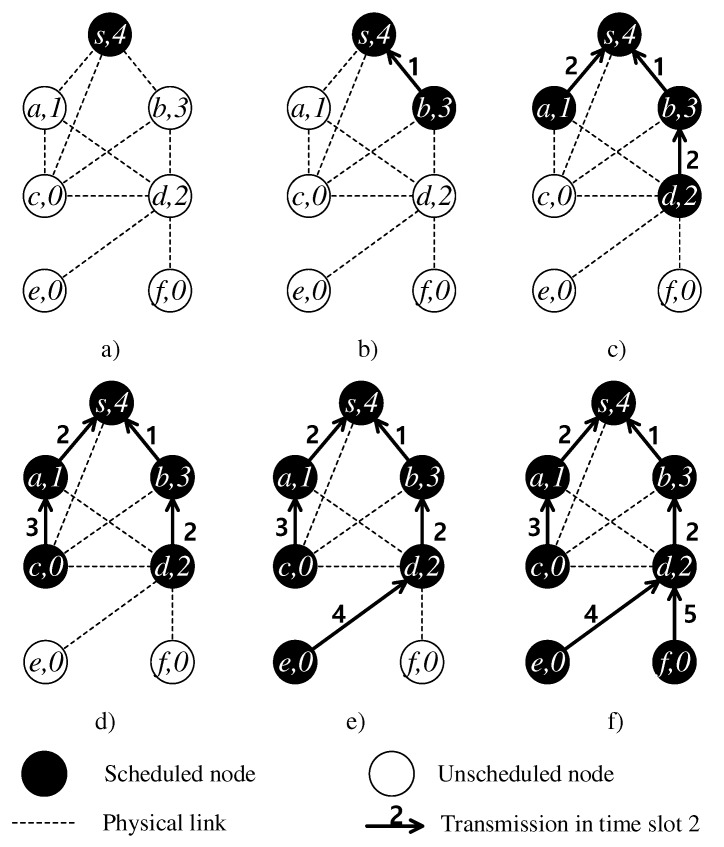
A step-by-step illustration of the proposed scheduling scheme.

Figure 6 shows a stepwise example of Algorithm 1 running on the communication graph Figure 6a. The set of scheduled nodes initially consists of the sink *s* only. In time slot 1, the candidate transmissions are {(a,s),(b,s)}. Their conflict degrees tie at 1 because their transmissions to the sink affect each other. Since mat(b)=3>mat(a)=1, node *b* gets the first time slot (Figure 6b). Then, link (a,s) will be removed from the candidate transmission set because it conflicts with the selected link (b,s). In time slot 2, the candidate parents are {s,b}. The candidate children are {a,c,d} as there exist communication links between them and the candidate parents. Now, the candidate transmission set is L={(a,s),(c,s),(c,b),(d,b)}. As analyzed in Figure 4, $l_{L}\left(a,s\right)=l_{L}\left(d,b\right)=2$ and $l_{L}\left(c,s\right)=l_{L}\left(c,b\right)=3$. The set of links with the smallest link conflict degree K={(a,s),(d,b)} and the algorithm must choose among {(a,s),(d,b)}. Since mat(d)=2>mat(a)=1, the link (d,b) is selected. Next, it removes (d,b) and its conflicted link(s) in *L*, i.e., {(c,s),(c,b)}, so L={(a,s)}. The link (a,s) can also take time slot 2. The result of time slot 2 is shown in Figure 6c. The algorithm continues until all the nodes are scheduled, as finally shown in Figure 6f.

## 5. Performance Evaluation

In this section, we evaluate the performance of our proposed scheme RADAS in terms of data aggregation time, which is the number of time slots needed since the first node transmits data toward the sink, until when the last transmission reaches the sink node. We present the methodology to carry out the simulation and the parameter settings in Section 5.1. Next, the reference schemes are listed in Section 5.2. After that, we measure the impact of our proposed metrics in Section 5.3. In Section 5.4, we evaluate the changes in the solution results under different network density and network size settings.

### 5.1. Simulation Settings and Methodology

We use same simulation settings as in [13,14]. The network consists of *n* static sensor nodes deployed in a two-dimensional square area (H×H). The sink is at the center of the area. The communication range of every node is equal and normalized to 1. The network density, which is the average number of neighbors within a disk area of radius 1, can be computed as follows [13]:

$$D=\frac{n\pi }{H^{2}}.$$

We will vary the network density in range 15–95 and network side length in range 1–8. When the network density *D* varies, H∈{2,4,7}. When the network side length varies, D∈{15,45,85}. For each case, we generate 30 random networks and collect the average results. Table 2 presents the simulation settings.

### 5.2. Baseline Schemes

We select a well known scheme in a sequential approach to compare: Minimum Lower bound Spanning Tree (MLST) and Neighbor Degree Ranking (NDR) scheduling algorithm [14]. MLST sums the hop distance to the sink and number of children up as the lower bound of the aggregation time, and uses it as the metric guideline for tree construction. Note that, by NDR, we mean including the supplementary scheduling algorithm in [14]. Such supplementary scheduling allows transmissions outside of the tree, which can further shorten the schedule length. The time to run MLST plus NDR is $O(|V{|}^{3}\Delta )$.

Regarding the simultaneous approach, we select the Greedy Growing Tree (GGT) scheduling scheme as a baseline. The GGT uses node-related metrics, i.e., node degree and node role (*articulation node, pilot node, critical node*), to organize the transmission order. For example, *articulation node* will make the network disconnected once being removed. Such node type is the sole forwarder for a number of other nodes, so that how many nodes depending on it determines its importance. In addition, because articulation nodes have a high priority, the nodes in the shortest path from an articulation one to the sink (called *pilot node*) are also classified to be special. Lastly, *critical nodes* also need to be scheduled early because removing a *critical node* from the network will cause a number of sensors to send data through a longer routing path to the sink. Their algorithm seems to be effective in very sparse networks, as there will be the presence of such special nodes. The complexity of constructing the GGT is $O(|V{|}^{3}\Delta^{2})$.

The evaluation schemes are listed in Table 3. The MLST-NDR and GGT denote the reference schemes in [10,14], respectively. RADAS-link, RADAS-node and RADAS are three variations of our proposed scheme by using the link-base, node-based, or both of them when scheduling. Details of how to implement such three variations are presented in Section 5.3.

### 5.3. Impact of Metrics

There are two metrics defined in this work, each of which contributes a certain effect to shorten the aggregation time. Here, we will evaluate the performance of the link-based and node-based metrics. RADAS is the main scheme that uses both link-based and node-based metrics in the sequence as described in Section 4. Let *RADAS-link* be the algorithm using only the link conflict degree metric. We obtain the RADAS-link by removing lines 5–7 from Algorithm 4; instead, the algorithm randomly chooses a link with smallest link conflict degree. Let *RADAS-node* be the algorithm that uses only the MAT metric to guide the scheduling process. We modify the **while** loop in Algorithm 4 as follows. The lines 4–7 are removed. Then, in place of those, firstly, it selects the sender with highest MAT: $u_{0}\leftarrow \underset{MAT(u)}{arg max}\left\{\left(u,v\right)\in D\right\}$. Secondly, it chooses a parent for the selected sender. Let $N_{C}\left(v\right)$ be the neighbor set of node v∈P that all the elements in the set are from *C*. The parent is $v_{0}$ with smallest $|N_{C}\left(v_{0}\right)|$, i.e., $v_{0}\leftarrow \underset{|N_{C}\left(v\right)|}{arg min}\left\{v\in P\cap N\left(u\right)\right\}$. The reason is that, the smaller $|N_{C}\left(v_{0}\right)|$ is, the smaller the number of conflicted links that would be removed in line 10. The rest of the loop is unchanged (lines 8–10).

**Figure 7:**
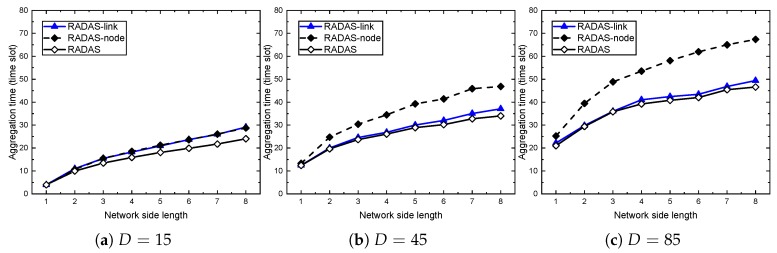
The impact of network side length on proposed schemes.

**Figure 8:**
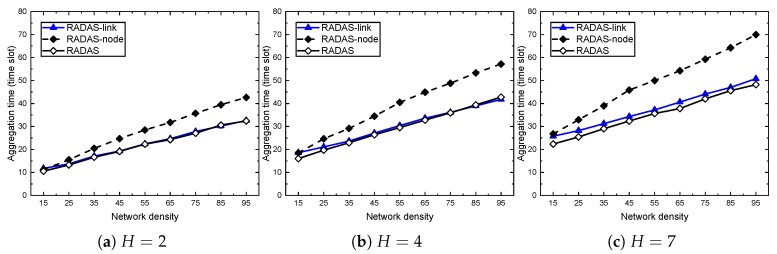
The impact of network density on proposed schemes.

The performance results are shown in Figure 7 and Figure 8. In each case, we vary the network side length and network density as indicated in the performance settings. Through all the cases, the RADAS-node takes the highest line, and the gap between RADAS-node and the two others are significant. At the smaller density network (D=15), RADAS offers about 16.5% shorter delay than the RADAS-link, but this ratio decreases as *D* increases. At D=45 and D=85, the improvements are up to 6.0–8.3%. The explanation is that, in an extremely high density network, the secondary collisions increase very fast, and it becomes a dominating factor compared to the primary collisions. As our node-based metric measures based on the primary collision model, its role becomes less effective at high density. On the other hand, we can see that, when the network side length is small (e.g., H=1), the aggregation time of the RADAS-link and RADAS schemes are almost the same. The RADAS lies slightly below the RADAS-link. This is reasonable because, with these settings, almost all the nodes are one hop away from the sink, so, based on the SPT constructed on the network, almost all of them have equal MAT. A similar trend happens when we keep the network side length constant, and vary the network density as in Figure 8.

As the RADAS holds the best performance record, in the next section, we will compare RADAS with the state-of-the-art solutions.

### 5.4. Comparison with Existing Solutions

**Figure 9:**
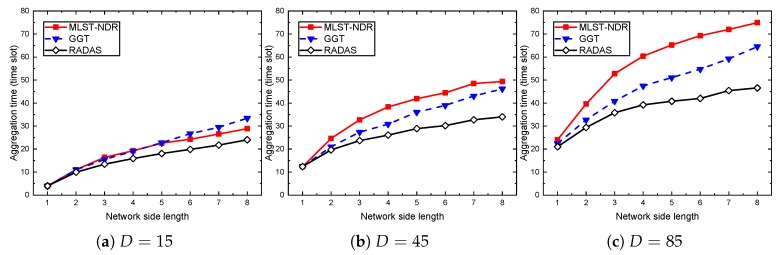
Aggregation time comparison when network side length varies.

**Figure 10:**
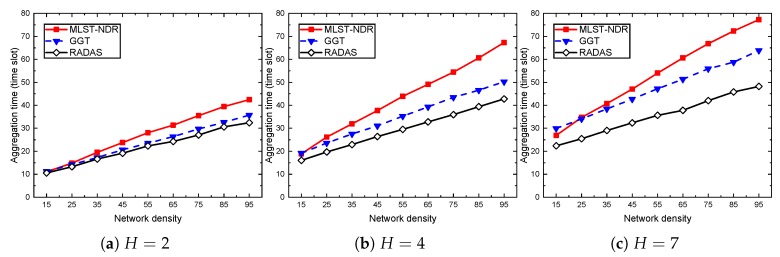
Aggregation time comparison when network density varies.

RADAS provides a decent improvement over the solutions in [10,14]. For example, in Figure 9, at D=85,H=8, the schedule length of RADAS is 38% shorter than [14] and 28% shorter than [10]. At D=45,H=5, the ratios are 31% and 20%, respectively. The aggregation times increase with the network side length as more nodes are present. Figure 10 shows the trend when the network density varies. When the network density gets higher, the amount of collisions that each transmission link causes to the surrounding area increases. As a consequence, it blocks an increased amount of other transmissions and lengthens the schedule. Our proposed scheme RADAS is consistently much better than the others, where the peak improvement is more than 25% at a high network density, compared with the reference schemes.

We intuitively clarify the reason behind such improvement as follows. In the scheme [14], when scheduling a node, NDR manages the transmission of the node to its parent in the MLST first. After filtering all the possible child–parent transmissions, the Supplementary Scheduling (SS) component of NDR starts to find alternative receivers for the unscheduled child nodes. Such SS mechanism supplies with up to 27% delay reduction compared to their non-SS one. However, because NDR gives the highest priority to tree-based links, which are determined without knowledge of secondary collisions, its outcomes, therefore, do not offer a good performance. On the other hand, GGT [10] uses a degree of each sender as a prioritization metric. However, degree of a node, in general, does not precisely reflect the conflict degree that a transmission from the sender may cause to the surrounding transmission candidates. In contrast, RADAS examines the conflict degree of every link with other transmission candidates. Selecting transmissions based on conflict degree could provide a larger set of concurrent transmissions at every time slot, which eventually leads to a smaller delay. Furthermore, RADAS uses a novel node metric, i.e., MAT, to relatively identify which nodes potentially have a big subtree. Those nodes are given higher priority since a big subtree requires more time to aggregate data. The effect of using MAT is proven in Section 5.3.

## 6. Conclusions

In this paper, we proposed a novel aggregation scheduling algorithm subject to minimizing the data aggregation delay in WSNs. We showed that the whole problem can be viewed as a maximum bipartite matching problem within each time slot. In that, we proposed and utilized both the link-based and node-based metrics to guide the scheduling process. While the former one finds a maximum number of concurrent transmissions in each time slot, the latter points out the transmission that can be beneficial for subsequent matching. We conducted an extensive simulation with various settings to observe the effect of link-based metric, and node-based metric alone or together. The results showed that our proposed idea significantly shortens the data aggregation delay, up to 30% compared to existing schemes. In addition, our algorithm can be an engine for a set of MLAS-derived problems. With some adjustments, the algorithm can be extended to perform well in multi-channel, duty-cycled or deadline-constrained, etc. data aggregation in WSNs. Not limited to those directives, in the future, we also plan to make a testbed to evaluate the performance of proposed scheme under real conditions, such as with the presence of unreliable links, as well as taking into account the energy-harvesting model.

## Figures and Tables

**Table 1:** Notation.

G=(V,E)	The modeled communication graph *G*, vertex set *V* and link set *E*
*s*	Sink node, s∈V
N(u)	Neighbors of node *u*
(u,v)	A link between the sender *u* and the intended receiver *v*
$l_{L}\left(u,v\right)$	The conflict degree of link (u,v) in the set of links L⊆E
p(u)	The parent of node *u*
C(u)	The child set of node *u*
mat(u)	Minimum Aggregation Time at node *u*
timeslot(u)	Transmitting time slot of node *u* to its parent avoiding all collisions

**Table 2:** Simulation parameters.

Name	Explanation
Network density (*D*)	15–95
Network side length (*H*)	1–8
Sink position	corner
Number of experiments	30

**Table 3:** Evaluation schemes.

Name	Explanation
MLST-NDR	running the NDR scheduling algorithm on MLST [14]
GGT	GGT scheme [10]
RADAS-link	our Reverse Approach for Data Aggregation Scheduling using link conflict degree
RADAS-node	our Reverse Approach for Data Aggregation Scheduling using MAT
RADAS	our Reverse Approach for Data Aggregation Scheduling using link conflict degree and MAT

## Appendix A

The following lemma will be used in the proof of Theorem 1.

**Lemma** **A1.**
*Let $t^{*}\left(u\right)=\max\left\{t\left(v_{i}\right)|v_{i}\in C\left(u\right)\right\}$. Then, $t^{*}\left(u\right)$ satisfies:*

(A1) $$t^{*}\left(u\right)\geq mat\left(v_{i}\right)+k-i+1,\;\mathrm{where}\;1\leq i\neq j\leq k.$$

**Proof** **of** **Lemma** **A1.** For any pair $\left(v_{i},v_{j}\right)$, from the inequations (1) and (2), we have:

(A2) $$t\left(v_{j}\right)>mat\left(v_{j}\right)\geq mat\left(v_{i}\right),1\leq i\leq k,i\leq j\leq k.$$

By definition of $t^{*}\left(u\right)$, from the inequation (A2), we can derive:

(A3) $$t^{*}\left(u\right)\geq t\left(v_{j}\right)>mat\left(v_{i}\right),i\leq j\leq k.$$

Since there are (k−i+1) nodes $v_{j}$ in the inequation (A3) with pairwise distinct transmitting time slots, the inequality (A1) holds. □

**Proof** **of** **Theorem** **1.** By definition, $t^{*}\left(u\right)$ can be seen as the aggregation time at node *u*. Since the inequation (A1) is true for every assignment of transmitting time, the minimum value of $t^{*}\left(u\right)$, i.e., mat(u) also satisfies the inequality:

(A4) $$mat\left(u\right)\geq mat\left(v_{i}\right)+k-i+1,1\leq i\leq k.$$

Next, we need to find the smallest value of mat(u). We will point out a valid assignment of transmitting time slots for the children of *u*, i.e., the inequations (2) and (3) are satisfied, given the Equation (4). Let’s consider the following transmitting time slot assignment:

(A5) $$t\left(v_{i}\right)=mat\left(u\right)-k+i,1\leq i\leq k.$$

Since the equation (A5) assigns a distinct time slot to each child, it always satisfies the inequation (3). From the inequation (A4) and Equation (A5), we have $t\left(v_{i}\right)\geq mat\left(v_{i}\right)+1$, which is equivalent to inequation (2). Theorem 1 is proved. □
